# Performance of Urinary Markers for Detection of Upper Tract Urothelial Carcinoma: Is Upper Tract Urine More Accurate than Urine from the Bladder?

**DOI:** 10.1155/2018/5823870

**Published:** 2018-01-30

**Authors:** Simone Bier, Jörg Hennenlotter, Michael Esser, Sarah Mohrhardt, Steffen Rausch, Christian Schwentner, Moritz Maas, Susanne Deininger, Simon Walz, Jens Bedke, Arnulf Stenzl, Tilman Todenhöfer

**Affiliations:** Department of Urology, Eberhard Karls University, Tuebingen, Germany

## Abstract

**Objectives:**

To assess the performance of urine markers determined in urine samples from the bladder compared to samples collected from the upper urinary tract (UUT) for diagnosis of UUT urothelial carcinoma (UC).

**Patients and Methods:**

The study comprised 758 urine samples either collected from the bladder (*n* = 373) or UUT (*n* = 385). All patients underwent urethrocystoscopy and UUT imaging or ureterorenoscopy. Cytology, fluorescence in situ hybridization (FISH), immunocytology (uCyt+), and nuclear matrix protein 22 (NMP22) were performed.

**Results:**

UUT UC was diagnosed in 59 patients (19.1%) (UUT urine) and 27 patients (7.2%) (bladder-derived urine). For UUT-derived samples, sensitivities for cytology, FISH, NMP22, and uCyt+ were 74.6, 79.0, 100.0, and 100.0, while specificities were 66.6, 50.7, 5.9, and 66.7%, respectively. In bladder-derived samples, sensitivities were 59.3, 52.9, 62.5, and 50.0% whereas specificities were 82.9, 85.0, 31.3, and 69.8%. In UUT-derived samples, concomitant bladder cancer led to increased false-positive rates of cytology and FISH.

**Conclusions:**

Urine markers determined in urine collected from the UUT exhibit better sensitivity but lower specificity compared to markers determined in bladder-derived urine. Concomitant or recent diagnosis of UC of the bladder can further influence markers determined in UUT urine.

## 1. Introduction

A high proportion of upper urinary tract (UUT) urothelial carcinomas (UC) (60%) are invasive at the time of diagnosis. The diagnosis of UUT UC, especially in early stages, is difficult and in many cases fortuitous. Typical symptoms like hematuria and flank pain are unspecific, and a lumbar mass is only detectable in 10–20% of all cases and reflects an advanced tumor stage [1, 2].

CT urography plays a decisive role for detecting UUT UC, but flat lesions are commonly undetectable by CT urography until the development of a urothelial thickening [2, 3]. The European guidelines recommend performing CT urography as a part of a standard diagnostic. Flexible ureteroscopy of the upper tract is considered as a major tool for diagnosis of UUT UC, and a ureteroscopic biopsy can determine tumor grade with a low false-negative rate [4]. Additional cystoscopy is required to rule out a UC in the bladder or the prostatic urethra [2].

Several studies have discussed the relevance of urine markers to detect UUT UC [3, 5, 6]. Still, urine cytology is the only marker, which is recommended by current guidelines, even if it is known to be less sensitive for the detection of UUT UC compared to UC of the bladder [2]. Due to its high specificity, a positive cytological examination always necessitates an additional endoscopic examination [2, 7]. It has been frequently discussed that the detection rate of UUT UC may be increased by analyzing urine markers in urine derived from the upper urinary tract instead of urine derived from the bladder (in most cases midstream urine) [7, 8].

Hence, aim of this study is to compare the performance of the four most broadly available markers cytology, fluorescence in situ hybridization (FISH), immunocytology (uCyt+), and nuclear matrix protein 22 (NMP22) when determined in urine derived from the bladder or directly from the UUT.

## 2. Patients and Methods

### 2.1. Patients and Samples

A total number of 758 urine samples of 682 patients with suspicion of UUT UC between 2004 and 2012 were retrospectively included into the study. Of 758 samples, 373 urine samples were collected from the bladder and 385 from the UUT. In 297 cases, UUT urine was collected using ureteronoscopy whereas ureteral catheters were used in 88 cases. In case contrast medium was applied, urine sampling was performed before application of contrast medium (if feasible). All patients underwent cystoscopy and UUT imaging or ureterorenoscopy. Patients with other malignant diseases than UC were excluded. In case of bladder-derived urine sampling, all patients with present BC in cystoscopy were excluded. In bladder-derived urine samples, cytology, FISH, immunocytology, and NMP22 ELISA were performed in 373, 282, 193, and 254 patients, respectively. In UUT-derived samples, cytology, FISH, immunocytology, and NMP22 ELISA were performed in 379, 171, 10, and 40 patients, respectively. The study was approved by the local ethics committee (number 400/2009A).

### 2.2. Urine Markers

Urine markers were performed as previously described [9–13]. Procedures were identical for bladder-derived and UUT-derived samples. Urine was centrifuged, cytospinned, and stained after PAP staining for cytology. Subsequent microscopic assessment was done according to the recommendations of the Papanicolaou Society of Cytopathology [9]. The UroVysion assay was considered positive after the manufacturer's recommendations as previously described [10]. The uCyt+ test was considered positive if at least one cell showed a positive signal of CEA or mucin [11, 13]. NMP22-ELISA was performed according to the manufacturer's protocol with a threshold for a positive test of 10 IU/mL [12].

### 2.3. Statistical Analysis

Statistical analysis was performed using JMP 7.2 (SAS Inc., Cary, USA) software. Contingency analyses were performed to determine sensitivities, specificities, and negative and positive predictive values.

## 3. Results

### 3.1. Patient Cohorts

We evaluated 385 UUT urine samples of 309 patients and 373 bladder-derived urine samples from 373 patients. Diagnostics including urine markers was applied due to hydronephrosis or other abnormalities in imaging in 245 (32.3%) and due to persistent microscopic hematuria in 116 patients (15.3%). In 127 patients (16.8%), markers were tested during surveillance of urothelial carcinoma of the lower urinary tract. 22 patients (2.9%) had recurring urinary infections and 31 (4.1%) patients recurring positive urinary markers. In 139 patients (18.3%), urine markers were performed as a result of irritative voiding symptoms or dysuria.

In the cohort of patients in whom UUT urine samples were available, 83 patients (26.8%) had a UC at the time of urine marker testing. Of these patients, 59 had a UUT UC (19.1%), 9 of these 59 patients had concomitant BC. Tumor stages and gradings are summarized in Table 1. Tumors were found in the renal pelvis of 29 patients (49.1%), in the ureter of 20 patients (33.9%), and in the renal pelvis and in the ureter of 7 patients (11.9%). In 3 patients, exact information on tumor location was not available (5.1%).

In the cohort of patients with urine samples derived from the bladder, 27 patients (7.2%) had a UUT UC at the time of diagnosis. In this cohort, patients with BC were excluded. Tumor stages and gradings of these patients are summarized in Table 1. Tumors were found in the renal pelvis, proximal ureter, and distal ureter in 9 (33.3), 11 (40.7), and 5 (18.5%) patients, respectively. In 2 patients, no exact information on tumor location as well as tumor grading was available (7.4%).

### 3.2. Performance of Urine Markers in UUT and Bladder-Derived Samples

Sensitivities, specificities, positive predictive values (PPV), and negative predictive values (NPV) of urine cytology, FISH, NMP22-ELISA, and uCyt+ in urine samples derived from the upper and lower urinary tract are summarized in Table 2.

Sensitivities of urine cytology, FISH, NMP22-ELISA, and uCyt+ in samples from the urinary bladder and the upper tract according to tumor stage and grade are summarized in Table 3.

### 3.3. Recent or Concomitant Bladder Cancer

In a subset of patients in whom urine markers were tested in samples from the upper tract, sampling was performed within eight weeks after diagnosis of bladder-located UC. To address the potential impact of concomitant (not yet resected at time of UUT sampling) or recent (completely resected) UC of the bladder on samples derived from the upper tract, the rates of positive tests in patients without evidence of UUT UC (therefore presumably “false-positive” tests) were compared (i) of patients with current BC, (ii) of those with recently resected BC, and (iii) of those of patients without any BC. Results are shown in Table 4.

## 4. Discussion

At the time of diagnosis, 60% of the UUT UCs are in an invasive stage of disease [3, 14]. In 70–80% of the patients with an UUT UC, micro- or gross-hematuria is one of the symptoms, but only a few patients with hematuria present an UUT UC or BC [2, 15]. For the diagnosis of UUT UC, noninvasive urine markers have become more and more important within the last years, although these markers may be affected by different extrinsic factors and are therefore controversially discussed [2, 3, 7]. However, the collection of urine markers from the UUT is associated with discomfort by the patients, especially if performed under local anesthesia [2]. In the present study, we therefore evaluated the impact of local origin in urine marker collection on performance of urinary markers for detection of UUT UC.

We observed that sensitivity of cytology was remarkably higher in UUT-derived urine samples compared to urine from the bladder.

In current guidelines, cytology, preferably collected from the UUT, is the only recommended marker for diagnosis and follow-up of UUT UC [2]. In our study, in the cohort of patients with urine collected from the lower urinary tract, sensitivity of cytology was 59.3%. This is comparable with previous studies showing a sensitivity of 29–59% [5, 7]; nevertheless, in the cohort with urine collected from the lower urinary tract, 42.2% of the patients had a pTa or pT1 tumor stage while in the cited studies, the majorities of the patients had a tumor stage > pT1. This is especially important because it has already been shown that in patients with high-grade tumors of the bladder, the sensitivity of cytology—as well as NMP22 and uCyt+—is higher than that in patients with low-grade tumors [16]. Therefore, the sensitivities of urine cytology in our study appear relatively high. In the cohort of patients with cytology determined in UUT urine samples, sensitivity was as high as 74.6%, which is high compared to previous studies reporting a sensitivity for UUT urinary cytology between 43 and 79% [17]. The results of our study therefore support the hypothesis that a UUT-derived cytology is superior with regard to sensitivity compared to a bladder-derived analysis. The increase in sensitivity was associated with a loss of specificity suggesting that the mechanical manipulation associated with upper tract sampling has an impact on morphology of urothelial cells.

For FISH, we also observed a higher sensitivity but lower specificity when performed in urine samples from the upper tract. For bladder-derived samples, performance of FISH was inferior compared to previous studies reporting sensitivities of FISH in bladder urine between 77 and 100% (with specificities of 95–100%) [3, 18, 19]. Recent studies by Mian et al. and Akkad et al. detected higher specificities for UUT FISH testing [8, 20]. One of the reasons for the higher numbers of false-positive results and lower specificity in our study may be the use of different interpretation criteria compared to the other studies. We used the interpretation algorithm suggested by the manufacturer. We could recently show that the results of the UroVysion strongly depend on the algorithm used for interpretation. Especially, the use of different cut-offs for tetraploid could improve specificity in bladder-derived urines [21]. This may also be the reason for the higher specificity that another group reached in 2014 for the FISH test in UUT-sampled urines (91.1%) [8, 22].

While sensitivity of the FISH test was obviously higher when performed on UUT urine, the specificity was inferior. Whether this is due to tetraploidic cells detected due to the stress applied to the urothelium by mechanical manipulation of the upper tract remains to be elucidated.

Both in bladder- and UUT-derived samples, we observed considerably low specificities for NMP22. It has been shown previously that the NMP-22-test is highly vulnerable for different extrinsic factors [23, 24]. The manipulative extraction of UUT urine seems to be an important extrinsic factor for this test leading to decreased specificity. Therefore, the use of NMP-22 as UUT urinary marker cannot be recommended. We also observed a lower specificity for bladder-derived urine samples compared to previous studies showing a specificity of 85% and a sensitivity of 58% (25% for low-grade and 92% for high-grade tumors) [16]. The reason for lower specificity in our study might be a relatively high proportion of samples collected after instrumentation or mechanical manipulation. In conclusion, our results show that NMP22 should not be performed in urine from the upper tract or urine derived via instrumentation due to a high false-positive rate. Clear exclusion criteria should be respected when using NMP22.

Although our cohort included only a low number of patients with uCyt+ performed in UUT-derived urine (*n* = 10), sensitivity in this small cohort was clearly higher compared to bladder-derived urine. We did not observe an obvious difference in specificity between bladder- and UUT-derived urine. Previous studies have shown a good performance of uCyt+ for UUT detection in both bladder- and UUT-derived urine. In a study performed by Lodde et al. including 32 patients, sensitivities of uCyt+ performed in bladder- and UUT-derived urine samples were 75% and 91% while specificities were 95 and even 100%, respectively [25]. One potential explanation for inferior sensitivity in bladder-derived samples in our study may be the high proportion of patients with lower tumor stages in our cohort. Of note, sensitivities and specificities of uCyt+ for detection of UUT tumors are similar to sensitivities and specificities in BC cohort, which have been reported to be 63.3%–84.9% and 62.0%–78.1%, respectively [16, 26, 27].

The subgroup of our collective showing recent or even current BC revealed interesting results showing higher rates of positive markers in UUT-derived urine in the absence of UUT tumors. However, FISH tests in UUT urine (with the used cut-off criteria) showed a higher specificity in patients with previous BC than UUT cytology and one may speculate that previous therapy affects cell morphology even after weeks.

What are the clinical implications of the results of this study? First, our study shows that the sensitivity of cytology and other urine markers for detection of UUT tumors can be enhanced by sampling urine directly from the upper tract. A high sensitivity is especially required in the context of disease surveillance. In patients without history of urothelial carcinoma but other signs suggestive of a tumor in the urinary tract (such as microscopic hematuria), markers should provide a high specificity [28]. In this context, the analysis of upper tract urine should be considered critically, as specificities are clearly lower compared to bladder-derived urine. However, more clinical data from patients undergoing surveillance of UUT UC is needed before recommending other markers than cytology.

There are some limitations in this present work to be stated: One main limitation of our study is that we examined the urine markers of two independent cohorts. Another limitation is that the anticipatory-positive value urine markers could not be addressed because follow-up data was not available. Especially for FISH, previous studies suggest a prognostic role of positive markers in patients with negative endoscopic workup [29]. Moreover, the number of patients with NMP22 or uCyt+ results available from UUT-derived urine was relatively small.

## 5. Conclusion

The type of urine sampling affects the performance of urine markers in the context of upper tract urothelial carcinoma. The analysis of urine markers determined in urine directly sampled from the UUT is associated with a higher sensitivity and a lower specificity compared to urine markers determined in urine samples from the bladder. Due to the observed differences in sensitivity, future studies comparing urinary markers, imaging studies, and endoscopy in patients undergoing surveillance of patients with UUT UC should include urine analysis of UUT-derived urine.

## Figures and Tables

**Table 1:** Tumor stages and grades of the cohorts with urine markers determined in UUT-derived urine and bladder-derived urine.

		Cohort with markers determined in UUT-derived urine, *n* (%)	Cohort with markers determined in voided urine, *n* (%)
T-stage	pTa	27 (45.7)	16 (27.4)
	pT1	12 (20.3)	4 (14.8)
	>pT1	14 (23.7)	5 (18.5)

Grade	G1	23 (39.0)	12 (52.2)
	G2	22 (37.3)	9 (39.1)
	G3	8 (13.6)	2 (8.7)

Cis		6 (10.2)	2 (8.7)^∗^

UUT = upper urinary tract; Cis = carcinoma in situ. ^∗^Concomitant Cis.

**Table 2:** Test performance characteristics of urine markers determined in bladder-derived and upper urinary tract- (UUT-) derived urine.

Test	Source	Tests performed (*n*)	Positive tests, *n*	Sensitivity	Specificity	PPV	NPV total
Cytology	Bladder	354	72	59.3	82.9	22.2	96.1
	UUT	379	151	74.6	66.6	29.1	93.4

FISH	Bladder	264	46	52.9	85.0	19.6	96.3
	UUT	171	90	79.0	50.7	16.7	95.1

NMP22	Bladder	240	164	62.5	31.3	6.1	92.1
	UUT	40	38	100.0	5.9	15.8	100.0

uCyt+	Bladder	181	57	50.0	69.8	10.5	95.2
	UUT	10	4	100.0	66.7	25.0	100.0

FISH = fluorescence in situ hybridization; NMP22 = nuclear matrix protein 22; uCyt+ = immunocytology; PPV/NPV = positive/negative predictive value.

**Table 3:** Sensitivities for detection of upper urinary tract urothelium carcinoma of urine markers determined in bladder-derived and upper urinary tract- (UUT-) derived urine dependent on tumor stage and tumor grade.

Sensitivity [%]	Source	Tumor stage		Tumor grading	
		Ta	T1–4	G1/G2	G3/Cis
Cytology	Bladder	43.8	88.9	18.2	92.3
	UUT	65.4	76.9	72.1	78.6

FISH	Bladder	36.4	80.0	28.6	100.0
	UUT	71.4	100.0	73.3	100.0

NMP22	Bladder	50.0	100.0	57.1	75.0
	UUT	100.0	100.0	100.0	100.0

uCyt+	Bladder	25.0	100.0	50.0	66.7
	UUT	100.0	100.0	100.0	100.0

FISH = fluorescence in situ hybridization; NMP22 = nuclear matrix protein 22; uCyt+ = immunocytology; Cis = carcinoma in situ.

**Table 4:** Specificities and false-positive rates for cytology and FISH in UUT-derived urine dependent on concomitant or recent urothelial carcinoma of the bladder.

Status	Cytology		FISH	
	Specificity	False-positive rate, %	Specificity	False-positive rate, %
No BC	69.4	30.6	53.6	46.4
BC treated within last eight weeks, currently no BC	33.3	66.7	60.0	40.0
Concomitant BC	50.0	50.0	31.8	68.2

FISH = fluorescence in situ hybridization; BC = bladder-located urothelium carcinoma; UUT = upper urinary tract.
